# Association between polysomnography-measured sleep parameters and cognitive impairment in elderly patients with depression

**DOI:** 10.3389/fpsyt.2025.1485127

**Published:** 2025-04-24

**Authors:** Jiaojiao Zhou, Jianyu Que, Yida Wang, Li Ren, Saina Zhang, Xianglin Ma, Yintai Fan, Qing’e Zhang, Xueyan Chen

**Affiliations:** ^1^ National Clinical Research Center for Mental Disorders & National Center for Mental Disorders, Beijing Anding Hospital, Capital Medical University, Beijing, China; ^2^ Xiamen Xianyue Hospital, Xianyue Hospital Affiliated with Xiamen Medical College, Fujian Psychiatric Center, Fujian Clinical Research Center for Mental Disorders, Fujian, China

**Keywords:** polysomnography, sleep, cognitive impairment, elderly patient, depression

## Abstract

**Objective:**

Limited research has explored the associations between sleep disturbances (SD) and cognitive impairment (CI) in elderly patients with depression, particularly by incorporating polysomnography (PSG) to assess sleep quality. This study was conducted to determine correlations between PSG-quantified sleep parameters and CI among individuals with late-life depression.

**Methods:**

65 elderly patients with depression were included in the study. The sleep status was assessed using PSG, while cognitive function was evaluated using the Mini-Mental State Examination (MMSE). The correlation between PSG-measured sleep parameters and cognitive function was analyzed.

**Results:**

CI was observed in 31 (47.7%) individuals. Depressed elderly patients with CI exhibited a shorter total sleep time (TST) compared to those without CI. Furthermore, their sleep efficiency (SE) was reduced as evidenced by shortened durations and proportions of N1 and N3. Conversely, the proportion of non-rapid eye movement (NREM) and N2 increased in this group. Additionally, both the duration and proportion of rapid eye movement (REM) were decreased. Spearman correlation analysis revealed a linear relationship between the MMSE score and various sleep parameters. However, in the multiple linear regression model, only the proportions of NREM exhibited a significant linear relationship with the MMSE scores.

**Conclusions:**

In elderly patients with depression, a significant linear relationship was observed between the MMSE score and various sleep parameters measured by PSG.

## 1 Introduction

Depression is the most prevalent mental disorder among older adults worldwide, affecting 7% of the global elderly population and contributing to 5.7% of years lived with disability in individuals aged over 60 years (1). The association between cognitive impairment (CI) and depression has been observed in recent years (2, 3). CI may represent a particularly significant aspect of depression from a practical standpoint (4). After reaching the age of 65, older adults who received an initial diagnosis of depression demonstrated a significantly heightened association with CI (OR = 6.65, p < 0.01) (5). Accumulated evidence underscores the pivotal role of systemic inflammation in both CI and depression (6). Furthermore, oxidative stress emerges as a shared mechanism underlying cognitive decline and depression (7). Consequently, the relationship between CI and depression exhibits complexity and bidirectionality.

The previous studies have demonstrated that the prevalence of sleep disturbances (SD) in patients with depression ranges from 60% to 90%, whereas the prevalence of SD among this patient population in China is reported as 85.22% (8). Dysregulation of monoamine neurotransmitters, reduced glutamate production in the brain, and disruption of sleep-wake rhythmicity by dysregulating the hypothalamic-pituitary-adrenal axis may underlie SD observed in patients with depression (9).

SD among older adults has consistently been linked to mental health issues and shown to be associated with cognitive decline (10, 11). In clinical practice, both CI and SD are associated with the progression of depression. SD and CI are prevalent non-affective manifestations in patients with depression (12). Shorter than recommended sleep is believed to reduce brain health and cognitive performance (13). It is important to emphasize that current knowledge is far from sufficient to understand the role of SD in elderly depressed patients with CI. SD can be a symptom, a risk factor, or an early marker in elderly depressed patients with CI. Gaining a more comprehensive understanding of the sequential two-mediator relationship between depression progression and SD in connecting depression and CI can provide valuable insights into the clinical significance of monitoring and managing mood and sleep among older adults, particularly highlighting the cognitive benefits of addressing late-life depression to mitigate CI.

In previous studies, researchers have employed a variety of methodologies to assess SD and CI. While the Pittsburgh Sleep Quality Index (PSQI) has commonly been utilized as a subjective tool, actigraphy devices have also been used. However, polysomnography (PSG), considered the gold standard for detecting SD (14), represents an objective and sensitive assessment of sleep status (15). To the best of our knowledge, limited research has explored the associations between SD and CI in elderly patients with depression, particularly by incorporating PSG to assess sleep quality. In non-rapid eye movement (NREM) stages N1, N2, and N3, a previous research suggested that slow wave sleep (SWS) is related to better cognitive function (16).Thus, there is a lack of research that has correlated neuropsychological test measures with PSG measurements (17). In light of this gap, we conducted this study to determine correlations between PSG-quantified sleep architecture disturbances and CI among individuals with late-life depression.

## 2 Materials and methods

### 2.1 Subjects

Participants were recruited from Beijing Anding Hospital between June 2021 and May 2024. The patients included in the study were diagnosed with depression based on the criteria outlined in the Diagnostic and Statistical Manual of Mental Disorders, 5th edition (DSM-5) criteria and Mini-International Neuropsychiatric Interview (M.I.N.I.). Eligible participants included individuals aged 60-85 years who exhibited moderate or greater severity of symptoms, as indicated by a score of ≥17 on the Hamilton Depression Scale (HAMD-17) (18). And they had not received antipsychotic treatment for at least 3 months. Patients with the following conditions were excluded: (1) Patients with a history of epilepsy or coronary heart disease or other serious unstable physical diseases; (2) visual and hearing loss, illiteracy, and the inability to complete the required tests; (3) a history of psychiatric diseases such as organic mental disorders, Alzheimer’s disease, secondary dementia of other causes, schizophrenia, schizoaffective disorder and bipolar disorder; (4) a history of substance abuse, including alcohol or active substance abuse within the past 12 months, except nicotine; and (5) use of medications that may affect cognition or sleep for nearly 2 weeks. Written informed consent was obtained from all participants. The study was approved by the Medical Ethical Committee in Beijing Anding Hospital of the Capital Medical University, China [(2021) Research No. (75)]. The demographic and clinical characteristics of each patient were systematically documented.

### 2.2 PSG examination

All the subjects were observed with overnight video PSG using a digital sleep laboratory system (Grael, Compumedics, Australia). All PSG sessions were meticulously supervised by a proficient technician in accordance with standardized criteria. The inter-rater reliability is regularly assessed and ensured to exceed 0.9 as an integral part of laboratory protocols. The following sleep characteristics were acquired and analyzed: total sleep time (TST); sleep efficiency (SE); the time and percentage of stage N1, N2, N3, NREM, and rapid eye movement (REM) sleep, and the apnea hypopnea index (AHI). Obstructive sleep apnea (OSA) is defined as the presence of an AHI ≥5/h.

### 2.3 Subjective sleep and neuropsychological evaluations

The PSQI, developed by Buysse et al. in 1989, was utilized to assess the subjective sleep statuses of all participants. This questionnaire consists of 19 items that evaluate seven subdomains including daytime dysfunction, sleep disturbance, subjective sleep quality, habitual sleep efficiency, sleep duration, sleep latency and hypnotics use on a 4-point scale (ranging from 0 to 3). The total score ranges from 0 to 21 points with higher scores indicating poorer sleep quality. Individuals scoring ≥5 are categorized as poor sleepers (19).

Hamilton Depression Rating Scale (HAMD‐17) were used to assess depressive symptoms, with a HAMD-17 score ≥ 24 indicating the presence of severe depression (20). Cognitive functioning was evaluated with the Chinese version of the Mini Mental State Examination (MMSE) (21). The MMSE comprises a variety of cognitive tasks, encompassing immediate and delayed word recall, animal naming, word recognition, symbol digit modalities test, immediate constructional praxis, digit span backwards test, immediate and delayed logical memory, number series and trail-making tests. These tasks collectively contribute to a maximum achievable score of 30 points. A MMSE score < 24 is indicative of CI (22, 23).

### 2.4 Statistical analysis

All statistical tests were performed with SPSS 26.0 software (IBM Corp, Armonk, NY, USA). Statistical significance was set as a p-value<0.05 (two-tailed). The Chi-square test was used to determine the distribution of categorical variables, and the Student’s t test or Mann-Whitney U test was used for continuous variables. The Spearman correlation analysis was employed to examine the correlation between PSG-measured sleep parameters and cognitive function. Only those variables that were identified as significant (p<0.05) in the univariate analysis were included in the multivariate analysis. The sleep parameters of the patients were included in the multiple linear regression model along with the patients’ educational level, degree of depression, and OSA. This allowed for a comprehensive examination of the independent effects of each sleep parameter on CI. Figures were created using GraphPad Prism Version 9 (San Diego, USA).

## 3 Results

### 3.1 Demographic and clinical characteristics of all participants

A total of 65 elderly patients with depression, comprising 24 males and 41 females, were included in the study. The median age of 67 years (interquartile range, [IQR]: 65- 70), with a mean body mass index (BMI) 25.2 ± 4.0 Kg/m^2^. The PSQI had a median score of 15 (IQR:10-18), while the AHI had a median value of 4.90/h (IQR: 0.45-11.65). Among these patients, CI was observed in 31 (47.7%) individuals based on a MMSE score <24, while senior high school education or above was reported by only 22(33.8%) patients. Additionally, severe depression was present in 17 (26%) patients, and OSA was diagnosed in 31 (47.7%) patients. **Table 1** summarizes the socio-demographic and clinical characteristics between CI and non-CI groups. The results revealed that CI was more prevalent among patients with severe depression (39.6% vs 70.6%, p=0.028), those with lower than senior high school education (22.7% vs 60.5%, p=0.004), higher PSQI scores (10 vs 17, p<0.001), or OSA (32.4% vs 64.5%, p=0.010). No significant differences were found regarding age, gender, BMI, and AHI when comparing subgroups.

**Table 1:** General demographic, clinical characteristics and PSG-measured sleep parameters of patients with and without CI.

Characteristics	Entire cohort	No CI	CI	Z or χ^2^	P-value
Age, years, M(IQR)	67 (65,70)	67 (66,70)	68 (64,69)	-0.587	0.557
Gender, n (%)				0.081	0.776
Male	24 (36.9%)	12 (50.0%)	12 (50.0%)		
Female	41 (63.1%)	22 (53.7%)	19 (46.3)		
Education, n (%)				8.308	0.004
≥Senior high school	22 (33.8%)	17 (77.3%)	5 (22.7%)		
<Senior high school	43 (66.2%)	17 (39.5%)	26 (60.5%)		
Depression status, n (%)				4.838	0.028
HAMD ≤ 24	48 (73.8%)	29 (60.4%)	19 (39.6%)		
HAMD>24	17 (26.2%)	5 (29.4%)	12 (70.6%)		
BMI, Kg/m^2^	25.2 ± 4.0	25.9 ± 3.7	24.4 ± 4.2	1.432	0.157
PSQI	15 (10,18)	10 (8,16)	17 (15,18)	4.362	<0.001
AHI,	4.90 (0.45, 11.65)	1.80 (0.38, 8.25)	5.80 (0.60, 21.50)	1.448	0.148
OSA, n (%)				6.724	0.010
No	34 (52.3%)	23 (67.6%)	11 (32.4%)		
Yes	31 (47.7%)	11 (35.5%)	20 (64.5%)		
TST, min	299.7 ± 80.3	323.2 ± 66.6	273.9 ± 86.9	2.579	0.012
SE, %	58.8 ± 15.3	63.6 ± 12.9	53.8 ± 16.3	2.698	0.009
NREM, min	269.2 ± 75.8	281.4 ± 67.8	255.9 ± 82.8	1.361	0.178
N1	35.8 ± 21.0	44.5 ± 21.3	26.2 ± 16.2	3.901	<0.001
N2	191.5 ± 52.4	183.8 ± 44.3	200.0 ± 59.7	-1.228	0.225
N3	41.9 ± 27.6	53.1 ± 28.7	29.7 ± 20.5	3.791	<0.001
NREM, %	89.7 ± 5.0	87.6 ± 5.3	92.1 ± 3.2	-4.167	<0.001
N1	11.5 ± 5.2	13.7 ± 5.6	9.0 ± 3.5	4.063	<0.001
N2	65.1 ± 11.6	58.0 ± 10.1	72.9 ± 7.3	-6.760	<0.001
N3	13.1 ± 6.7	15.9 ± 6.7	10.1 ± 5.3	3.812	<0.001
REM, min	30.6 ± 16.3	38.6 ± 16.8	21.8 ± 10.3	4.793	<0.001
REM, %	10.3 ± 4.9	12.3 ± 5.4	8.1 ± 3.2	3.900	<0.001

CI, cognitive impairment; IQR, interquartile range; HAMD, Hamilton Depression Rating Scale; BMI, body mass index; PSQI, Pittsburgh Sleep Quality Index; AHI, apnea hypopnea index; OSA, obstructive sleep apnea; PSG, polysomnography; TST, total sleep time; SE, sleep efficiency; NREM, non-rapid eye movement; REM, rapid eye movement.

### 3.2 PSG-measured sleep parameters of all participants

The average TST of the 65 patients was 299.7 ± 80.3 min, with an average SE of 58.8 ± 15.3%. And 64 (98.5%) patients suffered from SD with a SE < 85% (24). The mean NREM sleep duration was 269.2 ± 7 5.8 min, comprising N1 (35.8 ± 21.0 min), N2 (191.5 ± 52.4 min), and N3 (41.9 ± 27.6 min). The proportion of NREM sleep accounted for an average of 89.7 ± 5.0%, with N1 representing 11.5 ± 5.2%, N2 representing 65.l ± 11.6%, and N3 representing 13.l ± 6.7%. On average, REM sleep lasted for 30.6 ± 16.3 min, constituting 10.3 ± 4.9%. A comparison between the two groups regarding PSG-measured sleep parameters is presented in **Table 1**. The results revealed that depressed elderly patients with CI exhibited a shorter TST compared to those without CI (323.2 ± 66.6 min vs 273.9 ± 86.9 min, t = 2.579, p = 0.012). Furthermore, their SE was reduced (63.6 ± 12.9% vs 53.8 ± 16.3%, t =2.698, p = 0.009), as evidenced by shortened N1 and N3 sleep durations (44.5 ± 21.3 min vs 26.2 ± 16.2 min, t =3.901, p < 0.001; 53.1 ± 28.7 min vs 29.7 ± 20.5 min, t = 3.791, p < 0.001, respectively) and proportions (13.7 ± 5.6% vs 9.0 ± 3.5%, t = 4.063, p < 0.001; 15.9 ± 6.7% vs 10.1 ± 5.3%, t = 3.812, p < 0.001, respectively). Conversely, the proportion of NREM sleep and N2 sleep increased in this group (87.6 ± 5.3% vs 92.1 ± 3.2%, t = -4.167, p < 0.001; 58.0 ± 10.1% vs 72.9 ± 7.3%, t = -6.760, p < 0.001, respectively). Additionally, both the duration and proportion of REM sleep were decreased (38.6 ± 16.8 min vs 21.8 ± 10.3 min, t = 4.793, p < 0.001; 12.3 ± 5.4% vs 8.1 ± 3.2%, t = 3.900, p < 0.001, respectively). Notably, there were no significant differences observed in the NREM or N2 sleep durations between the two groups.

### 3.3 Correlation between PSG-measured sleep parameters and cognitive function

**Figure 1:**
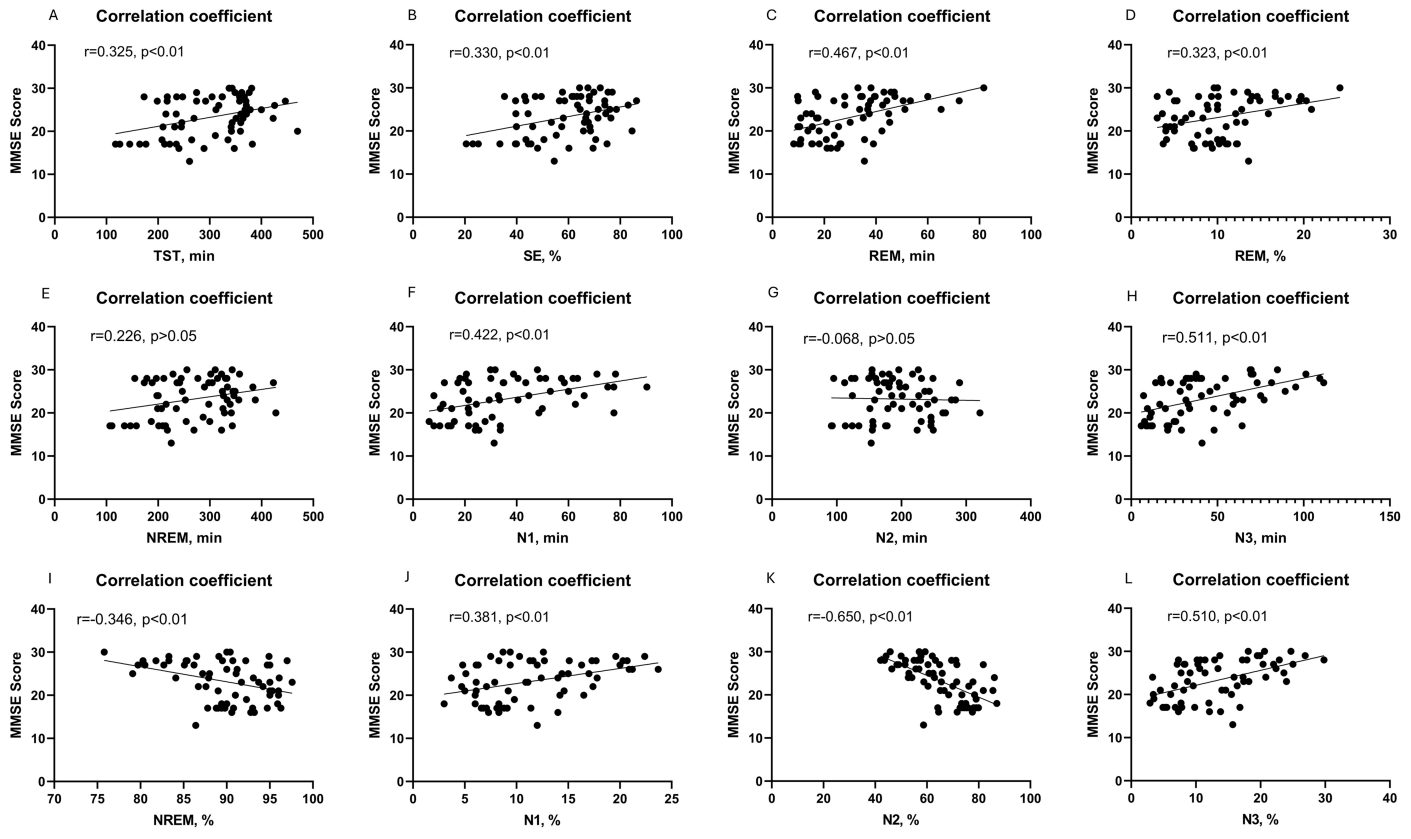
Correlation between cognitive function and PSG-measured sleep parameters, including TST sleep time **(A)**; SE **(B)**; REM sleep time **(C)**; REM sleep proportion **(D)**; NREM sleep time **(E)**; N1 sleep time **(F)**; N2 sleep time **(G)**; N3 sleep time **(H)**; NREM sleep proportion **(I)**; N1 sleep proportion **(J)**; N2 sleep proportion **(K)**; and N3 sleep proportion **(L)**.

The Spearman correlation analysis revealed a positive linear relationship between the MMSE score and various sleep parameters including TST (r = 0.325), SE (r = 0.330), N1 sleep time (r = 0.422), N1 sleep proportion (r = 0.381), N3 sleep time (r = 0.511), N3 sleep proportion (r = 0.510), REM sleep time (r = 0.467) and REM sleep proportion (r = 0.323) in patients (**Figures 1A–D, F, H, J, L**, respectively) (all p < 0.01). Conversely, there was a negative linear correlation observed between the MMSE score and the proportions of NREM (r = -0.346, p < 0.01) and N2 sleep (r = -0.650, p < 0.001) (**Figures 1I, K**). And there was no linear correlation between the MMSE score and NREM (r = 0.226, p > 0.05) or N2 sleep time (r = 0.068, p > 0.05) (**Figures 1E, G**). However, in the multiple linear regression model, only the proportions of NREM exhibited a significant linear relationship with the MMSE scores (p = 0.041) (**Table 2**).

**Table 2:** The associations between PSG-measured sleep parameters and CI in late-life depression were analyzed using multiple linear regression.

Characteristics	β	SE	β*	T	P-value	95% CI
Constant	126.018	51.785	–	2.433	0.018	22.104, 229.932
TST, min	0.015	0.036	0.258	0.416	0.679	-0.056, 0.086
REM, min	-0.001	0.181	-0.002	-0.004	0.997	-0.364, 0.363
REM, %	-0.889	0.747	-0.942	-1.190	0.239	-2.388, 0.610
N1, min	0.066	0.159	0.297	0.413	0.681	-0.253, 0.384
N3, min	0.027	0.095	0.159	0.282	0.779	-0.164, 0.217
NREM, %	-1.070	0.511	-1.143	-2.093	0.041	-2.096, -0.044
N1, %	-0.020	0.527	-0.022	-0.037	0.970	-1.076, 1.037
N3, %	0.113	0.320	0.163	0.352	0.726	-0.530, 0.756

PSG, polysomnography; SE, standard error; CI, cognitive impairment; CI, confidence interval; TST, total sleep time; SE, sleep efficiency; NREM, non-rapid eye movement; REM, rapid eye movement.

R^2^ = 0.502, adjusted R^2^ = 0.387, F = 4.371, p < 0.001.

*standardized.

## 4 Discussion

An increasing body of literature investigates the association between depression, sleep, and cognition; however, limited knowledge exists regarding older adults. Previous studies predominantly relied on questionnaires lacking objective evidence, which may raise concerns about recall bias and introduce uncertainty regarding participants’ actual sleep conditions (9). In our study, PSG was utilized to objectively evaluate sleep parameters in elderly patients with depression and their associations with cognitive impairment were examined. Among the 65 patients, CI was observed in 31 (47.7%) individuals. Depressed elderly patients with CI exhibited a shorter TST compared to those without CI. Furthermore, their SE was reduced as evidenced by shortened durations and proportions of N1 and N3. Conversely, the proportion of NREM and N2 increased in this group. Additionally, both the duration and proportion of REM were decreased. Spearman correlation analysis revealed a linear relationship between the MMSE score and various sleep parameters. However, in the multiple linear regression model, only the proportions of NREM exhibited a significant linear relationship with the MMSE scores. Those findings can shed light on the clinical relevance of monitoring and managing mental status and sleep for elderly patients with depression. These findings offer valuable insights into the clinical significance of monitoring and managing mental health status and sleep patterns in geriatric patients with depression.

Previous studies have demonstrated that the prevalence of sleep disorders in patients with depression ranges from 60% to 90% (8), which is comparatively lower than the observed rate of 98.5% reported in this study. This discrepancy may be attributed to the inclusion of exclusively elderly patients in our study cohort. Although aging per se does not lead to an escalation in SD, alterations in sleep architecture manifest with advancing age. A lot of empirical evidence suggests a high prevalence of SD within the elderly population (25, 26). In our study, the incidence of CI in elderly patients with depression was 47.7%, which was basically consistent with previous studies (27). Older adults with depression are prone to a progressive decline in mental health over time, which may contribute to age-related cognitive deterioration through pathogenetic mechanisms (28). This decline is characterized by an increased influence from subcortical emotion processing regions combined with attenuated top-down cognitive control (29). Recent studies have indicated that late-life depression also disrupts cognition through dysregulation of altered functional and structural brain connections (30).

A strong correlation has been observed between PSG-measured SD and CI in elderly patients with depression in our study. This is consistent with other studies that have shown impaired memory function due to disrupted sleep, either through a decreased proportion of REM sleep or reduced SE (31). In a previous study, the association between sleep duration and cognitive function was examined in a cohort of 1844 community-dwelling women; it was found that women who slept ≤5 h/night performed worse cognitively compared to those who slept 7 h/night (32). In NREM stages N1, N2, and N3, research focuses on the effects of SWS on cognition; results suggest that SWS is related to better cognitive function (16). In contrast to a previous study that, after controlling for multiple potential confounders, reported a U-shaped association between sleep duration and cognitive decline (33), our study was able to confirm only the declining trend in cognitive function with shorter sleep durations. This discrepancy may be attributed to the fact that all TST in our study were not higher than 10 hours. The reason for this phenomenon also might be Aβ deposition. Shorter sleep duration has been associated with increased Aβ deposition, indicating that SD contributes to CI rather than being solely a consequence of brain lesions. Moreover, Aβ accumulation in the regions involved in sleep regulation may exacerbate SD, leading to further elevation and accumulation of Aβ (34). In cognitively healthy older adults, both reduced sleep quality and shorter sleep duration have been linked to increased Aβ burden (35). Additionally, poor objectively measured sleep quality correlates with decreased CSF Aβ levels in healthy aging individuals (34). Earlier research using a mouse model of amyloidosis, which naturally develops Aβ plaques, demonstrated that extended periods of wakefulness were associated with higher ISF Aβ concentrations, whereas sleep deprivation hastened the formation of Aβ plaques (36, 37). Collectively, these findings suggest a potential connection between sleep and CI. Additional work is needed to determine whether these biological pathways mediate the association between sleep and cognitive function.

In contrast to other studies, we were not able to detect an influence of AHI on cognition. This might be due to the selection bias of patients with only very low AHI in this population, which is not representative for sleep disordered breathing patients. And we did not detect an association between cognition and BMI, underlining the assumption that the pathophysiology of cognition seems to differ between depression patients and the general population, which means that the clinical features pointing toward cognitive impairment in the general population seem not to be helpful in identifying patients at risk in the late-life depression population (38).

Several limitations should be acknowledged in this study. Firstly, PSG sleep tests were conducted without prior acclimatization of participants to the sleep laboratory, potentially introducing the first-night effect (i.e., differences observed in the initial PSG sleep recording compared to subsequent ones) (39), which has been shown to have a greater impact on older individuals than younger ones. However, it is less likely that this hypothesis holds true as the influence of the first-night effect on sleep structure would be expected to be similar in both cognitively impaired and non-impaired patients. Secondly, this work is limited by its relatively small sample size, which only allows for a limited number of statistical sub-analyses and lacks sufficient statistical power. Finally, the inclusion of certain confounding variables, such as socioeconomic status, was not accounted for in our study and should be considered as covariates in future research endeavors.

In conclusion, in elderly patients with depression, a significant linear relationship was observed between the MMSE score and various sleep parameters measured by PSG. Decreased sleep parameters, including TST, SE, duration of N1 stage, duration of N3 stage, proportion of N1 stage, and REM sleep time, along with an increased proportion of N2 stage, were identified as independent risk factors for CI.

## Data Availability

The raw data supporting the conclusions of this article will be made available by the authors, without undue reservation.
